# Oxidative and Inflammatory Markers Are Higher in Full-Term Newborns Suffering Funisitis, and Higher Oxidative Markers Are Associated with Admission

**DOI:** 10.3390/children9050702

**Published:** 2022-05-10

**Authors:** Michi Kamei, Mohamed Hamed Hussein, Ayako Hattori, Marwa Saleh, Hiroki Kakita, Ghada Abdel-Hamid Daoud, Akio Ishiguro, Fumihiko Namba, Makoto Yazaki, Haruo Goto, Ineko Kato, Hisanori Sobajima, Kabe Kazuhiko, Koichi Moriwaki, Hajime Togari

**Affiliations:** 1 Department of Pediatrics and Neonatology, Graduate School of Medical Science, Nagoya City University, Nagoya 467-8601, Japan; mkamei@med.nagoya-cu.ac.jp (M.K.); aykhat@med.nagoya-cu.ac.jp (A.H.); 2 Department of Medical Research and Development, Kinjo Gakuin University, Nagoya 463-8521, Japan; ikato@kinjo-u.ac.jp (I.K.); togari@kinjo-u.ac.jp (H.T.); 3 Division of Neonatology, Department of Pediatrics, Center of Maternal, Fetal and Neonatal Medicine, Saitama Medical Center, Saitama Medical University, Kawagoe 350-8550, Japan; marwa.saleh.smc@gmail.com (M.S.); akio-i@k4.dion.ne.jp (A.I.); nambaf@saitama-med.ac.jp (F.N.); sobajima@saitama-med.ac.jp (H.S.); kkabe_n@saitama-med.ac.jp (K.K.); 4 Department of Perinatal and Neonatal Medicine, Aichi Medical University, Nagakute 480-1195, Japan; hkaki1019@yahoo.co.jp; 5 Independent Researcher, AlMaadi, Cairo 11742, Egypt; ghada.a.daoud@gmail.com; 6 Department of Pediatrics, Hoshigaoka Maternity Hospital, Nagoya 464-0026, Japan; cbyazaki@gmail.com; 7 Department of Pediatrics, Nagoya City University West Medical Center, Nagoya 462-0033, Japan; gengotoapr@yahoo.co.jp; 8 Department of Pediatrics, Center of Maternal, Fetal and Neonatal Medicine, Saitama Medical Center, Saitama Medical University, Kawagoe 350-8550, Japan; kmoriwa@saitama-med.ac.jp

**Keywords:** neonate, free radicals, cytokines, inflammation

## Abstract

The aim of this study was to assess whether oxidative and inflammatory mediators in the cord blood of newborns with funisitis and chorioamnionitis can serve as indicators of their inflammatory status, and whether there is a positive association between higher mediator levels and an increased risk of admission to the neonatal intensive care unit (NICU). This study was conducted prospectively in a neonatology department of a university hospital. In total, 52 full-term newborns were evaluated, including 17 funisitis cases, 13 chorioamnionitis cases, and 22 control newborns without funisitis or chorioamnionitis. Cord blood samples were measured for oxidative stress and inflammatory status markers. The oxidative stress markers included the total nitric oxide (NO), total hydroperoxide (TH), biological antioxidant potential (BAP), and TH/BAP ratio, comprising the oxidative stress index (OSI). Inflammatory markers included interleukin (IL)-1b, IL-6, IL-8, IL-10, tumor necrosis factor alpha (TNFα), interferon γ (IFNγ), and complement component C5a. TH, OSI, IL-1b, IL-6, and IL-8 concentrations were higher in the funisitis group than in the chorioamnionitis and control groups. C5a was higher in the funisitis and chorioamnionitis groups than in the control group. Among all enrolled newborns, 14 were admitted to the NICU. Multiple logistic regression analysis showed that elevated umbilical cord blood levels of OSI and TH were associated with a higher risk of admission to the NICU (OSI: R = 2.3, 95% CI 1.26–4.29, *p* = 0.007 and TH: R = 1.02, 95%CI = 1.004–1.040, *p* = 0.015). In conclusion, OSI and TH in cord blood from full-term newborns can provide an index of inflammatory status, and higher levels are associated with the risk of admission to the NICU and, therefore, could serve as an early indicator of inflammatory conditions in newborns.

## 1. Introduction

The mediators of the fetal response to inflammatory conditions have been widely studied. Both funisitis (FN) [1] and chorioamnionitis (CAM) were reported to show an association with intrauterine infection and a higher incidence of preterm deliveries, which are both risk factors for maternal and neonatal morbidity and mortality [2,3].

The gold standard for evaluating antenatal inflammatory processes is the histopathological examination of the placenta. In amniotic infections, such as chorioamnionitis and funisitis, cytokines elicit a response from adjacent maternal leukocytes in membranes, the placental surface, and fetal leukocytes in the umbilical cord and in the wall of chorionic vessels present on the fetal surface of the placenta [4]. In hematogenous infections, the infectious agent produces a response that is first observed in intervillous spaces, placental villi, or decidua [3,4]. However, the pathological identification of chorioamnionitis and funisitis requires time in order to provide information for clinical care. Biomarkers could be more applicable to clinicians.

Intra-amniotic infections, such as funisitis or chorioamnionitis, are associated with higher levels of inflammatory cytokines, such as tumor necrosis factor alpha (TNFα) and interleukins (IL), including IL-1beta (IL-1b), IL-6 [5], and IL-8 [6] in amniotic fluid, cord blood, and newborn blood samples [7].

Inflammatory cytokines TNFα, IL-1b and IL-6, released into amniotic fluid or umbilical cord blood during the course of intrauterine infection in preterm infants, are associated with neonatal morbidity and long-term health impairment, such as bronchopulmonary dysplasia [8], brain lesions associated with white matter periventricular leukomalacia [9] and neonatal sepsis [10]. Furthermore, several infectious stimulators induce trophoblasts, microglia, and neutrophils to produce free radicals and nitric oxide [11]. Meanwhile, in full-term infants cord blood cytokines were shown to be associated with labor events [12].

The anaphylatoxin complement component C5a was reported to be elevated in neonatal sepsis hours before elevation in routinely measured markers of infection, including leukocyte count, differential blood count, and C-reactive protein [13]. Therefore, serum C5a concentration was suspected to be helpful in the early identification of severe systemic infection in newborns [13].

In addition, several reports have suggested that oxidants play a significant role in preterm labor, the pathogenesis of neonatal sepsis, and associated complications [14]. Antioxidant therapy is suspected to be useful in the treatment of newborns suffering from sepsis [15,16]. Oxidative stress and reactive oxygen species (ROS) play a crucial role in the induction and progression of various diseases in newborns, ranging from respiratory distress to sepsis in humans and septic shock in the neonatal sepsis model [17,18,19]. Oxidative stress can be quantified by measuring total hydroperoxide (TH), a measure of overall oxidative injury, because hydroperoxides are the product of lipid, peptide, and amino acid oxidation [20]. Furthermore, TH has been shown to be useful in identifying subjects at risk of hypoxia-induced oxidative stress [21,22,23]. Likewise, we have shown that TH surges in a neonatal sepsis model [19]. Therefore, the measurement of TH provides fundamental information on oxidative stress in critically ill newborns [21,22,23].

In contrast, the biological antioxidative potential (BAP) has been used to determine the overall antioxidative activity. Measuring BAP provides a reliable evaluation of the antioxidant barrier against oxidation, and suppressed oxidant potential due to exposure to ROS is a sign of many adverse conditions [19,21]. Oxidative stress develops when homeostasis between ROS formation and elimination by scavenging endogenous antioxidants is disrupted by TH overproduction or inadequate antioxidant defenses, detected by low BAP. The ratio between TH to BAP is used to calculate the oxidative stress index (OSI), an indicator used to evaluate balanced homeostats.

Oxidative stress markers and inflammatory cytokines have rarely been quantified in infants diagnosed with an intrauterine infection state or a normal placental histology status, respectively. The clinical application of these early biomarkers is still unclear for infants admitted to neonatal intensive units. This study aimed to investigate the association of these cord blood biomarkers with infant prenatal intra-uterine inflammation status and postnatal clinical condition at birth.

We measured the concentrations of TH, BAP, NO, cytokines, and C5a concentrations in the umbilical cord blood of newborns to determine whether their expression differs in the blood of newborns on their first day of life, diagnosed later on by histological examination of their placentas with funisitis and chorioamnionitis, and whether higher levels of these measured oxidative and inflammatory markers were associated with an increased risk of the admission of newborns to the NICU.

## 2. Materials and Methods

### 2.1. Patients

The study included 52 full-term newborns (gestational age 37–42 weeks) delivered at Nagoya City University West Medical Center between November 2006 and March 2008. Consent from the parents was obtained in all cases and the study was approved by the ethics committees of the West Medical Center of Nagoya City University and the Nagoya City University Hospital.

The placentas and umbilical cords of all the patients were histologically diagnosed with chorioamnionitis and/or funisitis. All further study measurements were performed in the Departments of Pediatric and Neonatology of Nagoya City University and the Saitama Medical Center, Saitama Medical University. Samples from infants that were small for gestational age, post-term infants, and newborns from mothers with inflammatory conditions other than infections were excluded.

### 2.2. Samples

Immediately after delivery, 3 to 5 mL cord blood samples were collected aseptically with sterilized sampling tubes, centrifuged at 3000 rpm for 10 min, and stored at −20 °C. All samples were tested to measure TH, BAP, NO, and the following cytokines: TNFα, IFNγ, IL-1b, IL-6, IL-8, IL-10, and C5a.

### 2.3. Histological Classification

In all cases, the placental discs and umbilical cords were separated after delivery. Sections were stored in 10% buffered formalin for histological examination. Each microscopic section was prepared in tissues embedded in paraffin and stained with hematoxylin–eosin before histological diagnoses were made. Histological diagnoses of chorioamnionitis and funisitis were determined by the degree of neutrophilic infiltration in stages I, II, and III based on the Blanc classification and the Nakayama classification for each, respectively [3,4,5].

**Figure 1:**
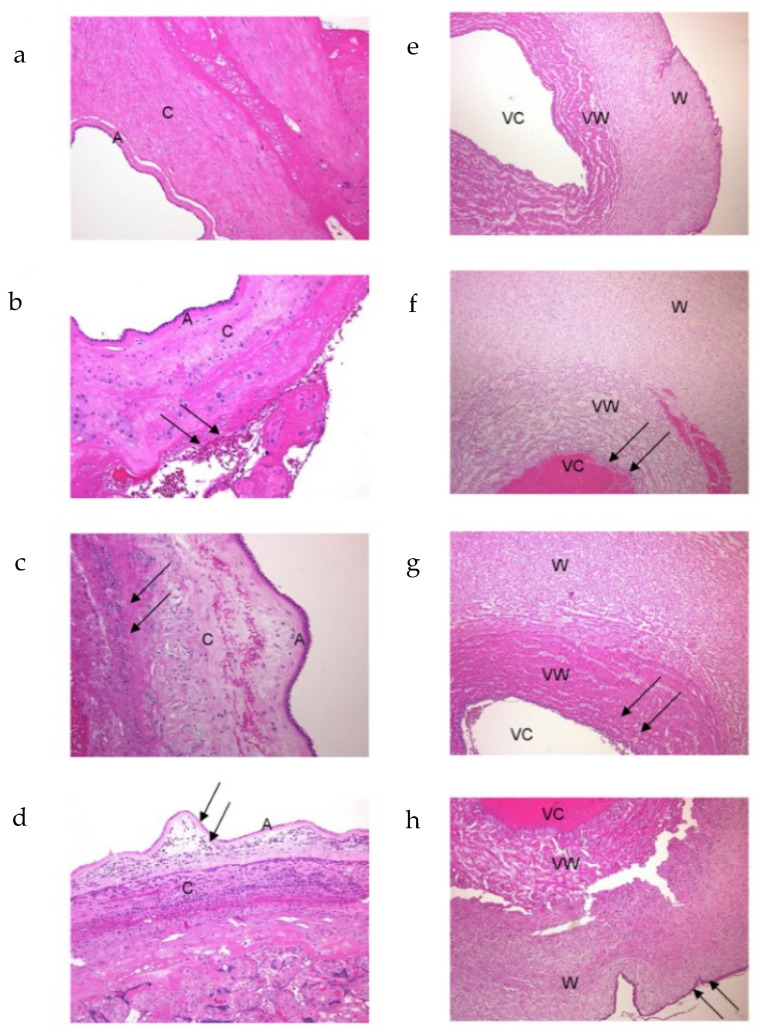
Compared with normal amniotic membranes (**a**), in cases of chorioamnionitis (CAM), maternal leukocytes can be seen to infiltrate membranes, while in cases of funisitis (FN), fetal leukocytes infiltrate and migrate in Wharton jelly. In CAM cases, neutrophils infiltrate the chorionic and amniotic membranes in the following steps: cells are first confined to the subchorionic area (stage I: **b**) (arrow); they then migrate to the chorionic membrane (stage II: **c**) (arrow); finally, they invade the amniotic membrane and the amniotic space (stage III: **d**) (arrow). The polymorph migration that occurs in umbilical cord vessels frequently starts in the vein and then moves to the arteries. Maternal neutrophils from villi spaces and neonatal-origin cells from umbilical vessels migrated to the chorionic membrane and the amniotic membrane placenta. A: amniotic membrane and C: chorionic membrane. Compared with the normal umbilical cord (**e**), in FN, the following were visible: stage I (**f**), migration and invasion of the vascular walls (stage II: **g**) (arrow), and infiltration of Wharton’s jelly (stage III: **h**) (arrow). VC: vascular cavity, VW: vascular wall, and W: Wharton’s jelly area. Magnification: 40× in (**a**–**d**) and 100× in (**e**–**h**).

The classifications of the histological stages of chorioamnionitis and funisitis are shown in Figure 1.

In cases of chorioamnionitis, maternal neutrophils were found to infiltrate the chorionic membrane with or without infiltrating the amniotic membranes of the placenta. Polymorph migration, which occurs in umbilical cord vessels, frequently starts in the veins and then progresses to the arteries. Maternal neutrophils from villi spaces and neonatal cells from umbilical vessels were observed infiltrating the chorionic membrane and the placental amniotic membrane, as previously reported by histological samples of the umbilical vein in each stage of funisitis (Figure 1).

### 2.4. Methods

#### 2.4.1. Serum Total Hydroperoxide (TH) Measurement

For this study, a small volume (10 µL) from each patient’s serum sample was used. Using a diacron reactive oxygen metabolites kit (d-ROM) (Diacron International srl, Proma, Italy) with the Free Radical Analytic System, the TH production was measured, as previously described [23].

#### 2.4.2. Serum Biological Antioxidative Potential (BAP) Measurement

Using a commercially available assay kit (Diacron) with the Free Radical Analytic System, the BAP was measured. In the BAP test, a small serum sample volume (10 μL) was dissolved in a colored solution obtained by mixing a source of ferric ions (ferric chloride, FeCl_3_) with a special chromogenic substrate (thiocyanate derivative). After an incubation period of 5 min at 37 °C, the colored solution occurs. The intensity of the color change is directly proportional to the ability of the serum sample to reduce the number of ferric ions to ferrous ions during incubation. These reduced ferric ions can be evaluated by the photometric assessment of the intensity of decolorization, and, therefore, an effective measurement of serum reduction ability or its so-called antioxidant potential is achieved [19,23].

#### 2.4.3. Calculation of the Serum Oxidative Stress Index (OSI)

The TH-to-BAP ratio gives OSI, because oxidative stress is defined as a shift toward the oxidative direction in the oxidative/antioxidant balance [16,19,23].

#### 2.4.4. Serum Total NO Concentration Measurement 

Total nitrite and nitrate (NO_2_− + NO_3_− [NO_x_]) in serum was measured by the Griess reaction using the Nitric Oxide Assay Kit (R&D Systems, Inc., Minneapolis, MN, USA) for measuring nitric oxide metabolites.

#### 2.4.5. Serum Cytokines Measurement

Serum cytokine levels of TNFα, IL-1b, IL-6, IL-8, IL-10, IL-12, and IFNγ were analyzed using the Cytometric Bead Array (CBA) system through FACS Calibur (BD Biosciences, San Diego, CA, USA). Each serum sample was measured to determine the concentrations of several cytokines according to the previously published method [24].

#### 2.4.6. Serum C5a Measurement

Using the BD OptEIA human C5a ELISA kit II (BD Biosciences, San Jose, CA, USA), which is specific for human C5a-desArg, serum C5a was evaluated. A reliable measurement of the level of complement activation that has occurred could be obtained by quantifying C5a-desArg in the serum samples [25].

### 2.5. Statistical Analysis

Data distributions were analyzed using the Shapiro–Wilk test. The means of the measurements of all three groups (intergroup means) were compared using analysis of variance (ANOVA), followed by the Bonferroni post hoc test. When the data were not normally distributed, the Kruskal–Wallis test was used, and when significance was detected, the Mann–Whitney test was used.

Cross-table analysis was performed using the chi-square test, and the coefficient of relationship was studied using the Spearman two-tailed test. Among all infants included in the study, the mean measurements of infants which require admission to the NICU after observation were compared with the measurements in infants that did not require admission using the unpaired student’s *t* test. When the data were not normally distributed, the Mann–Whitney test was used. A multivariate logistic regression model was also performed to examine the relationship of the level of each cord blood marker with admission to the NICU after adjusting for baseline variables. Variables with a *p* value < 0.05 on the bivariate analyses were included in the logistic regression analysis. Probability values less than 0.05 were considered significant. Data are expressed as mean ± standard error of the mean (SEM) unless otherwise noted. All data analyses were performed with the commercially available statistical analysis software package SPSS 28 (Statistical Package for Social Sciences, Chicago, IL, USA).

## 3. Results

### 3.1. Histological Classification

The classifications of the histological stages of chorioamnionitis and funisitis are shown in Figure 1. In cases of chorioamnionitis, maternal neutrophils were found to infiltrate the chorionic and amniotic membranes of the placenta. Polymorph migration, which occurs in umbilical cord vessels, frequently starts in the veins and then progresses to the arteries. Maternal neutrophils from villi spaces and neonatal cells from umbilical vessels were observed infiltrating the placenta of the chorionic membrane and the amniotic membrane, as previously reported by histological samples of the umbilical vein in each stage of funisitis, as shown in Figure 1.

### 3.2. Population Studied

**Figure 2:**
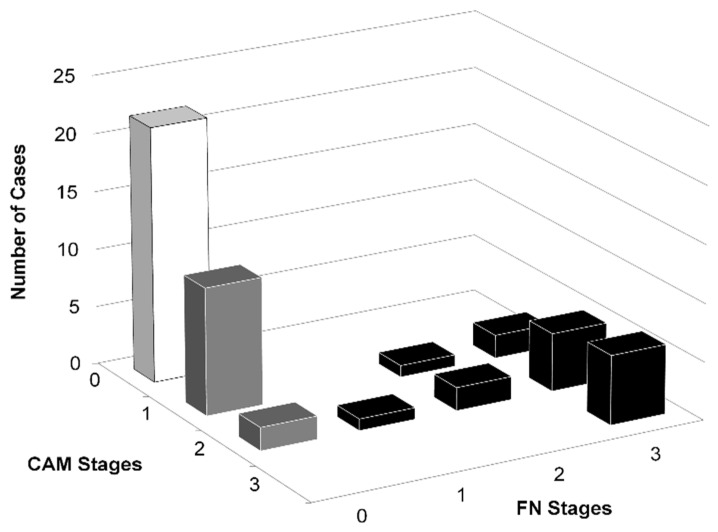
We studied a total of 52 newborns. This study group included 22 healthy newborns without funisitis (FN) or chorioamnionitis (CAM) as a control group (white column), 17 cases of FN (black column), and 13 cases of CAM (gray column). All members of the FN group suffered from CAM, while no member of the CAM group suffered from FN. A histological diagnosis of FN or CAM was made based on the degree of neutrophilic infiltration in stages I, II, and III using the Blanc classification [4].

The total number of newborns examined was 52. The groups consisted of 22 controls, 13 cases of chorioamnionitis, and 17 cases of funisitis. The 13 cases of chorioamnionitis were determined to not have funisitis. On the other hand, all the funisitis cases were associated with chorioamnionitis. Control newborns were diagnosed as free of funisitis and chorioamnionitis (Figure 2).

### 3.3. Characteristics and Clinical Courses of the Patients Studied

The clinical characteristics of the three groups are shown in Table 1. The gestational age of all subjects was between 37 and 42 weeks. Their birth weight ranged from 2466 to 3770 g. No significant differences were detected in gestational age, birth body weight, or gender between the funisitis, chorioamnionitis, and control groups.

An extended duration of premature membrane rupture (PROM) and markers of maternal infection, such as elevated C-reactive protein and elevated body temperature (above 38 °C), were more frequently observed in the funisitis group than in the control and chorioamnionitis groups (data not shown).

Apgar scores at 1 and 5 min did not show any significant differences between the groups. C-reactive protein levels in umbilical cord blood were higher in the funisitis group than in the control and chorioamnionitis groups, *p* < 0.001.

The admission rate to the neonatal intensive care unit (NICU) was higher in the funisitis group than in the control and chorioamnionitis groups, but there was no significant difference in admission rates between the chorioamnionitis and control groups (Table 1).

The indication of cesarean section and the indication of admission to the NICU are presented in Table 2.

### 3.4. Comparison among Funisitis, Chorioamnionitis, and Control Groups

#### 3.4.1. Oxidative Markers

**Figure 3:**
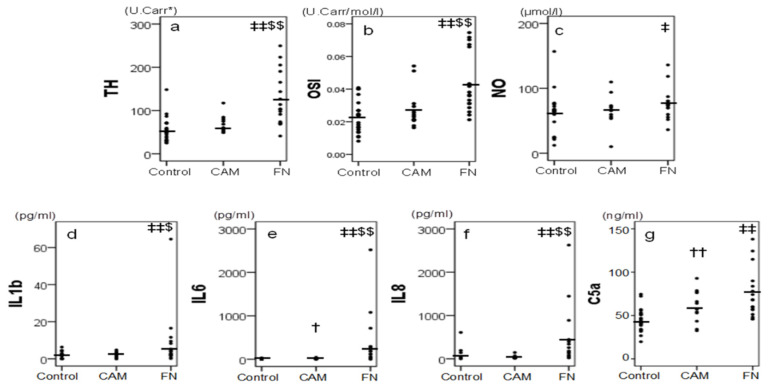
Serum cord blood levels of oxidative markers (**a**) TH, (**b**) OSI (TH/BAP) and (**c**) NOx, cytokines (**d**) IL-1b, (**e**) IL-6, (**f**) IL-8, and (**g**) C5a in 52 newborns: 22 healthy newborns without funisitis (FN) or chorioamnionitis (CAM) as a control group, 17 cases of FN, and 13 cases of CAM. † and ††: significant difference between control and CAM, *p* < 0.05 and 0.005, respectively, ‡ and ‡‡: significant difference between control and FN, *p* < 0.05 and 0.005, respectively, and $ and $$: significant difference between CAM and FN, *p* < 0.05 and 0.005, respectively. * The results of the TH are expressed in arbitrary conventional units, called Carr units, which are equal to a concentration of 0.08 mg/dL of hydrogen peroxide.

TH and OSI concentrations were significantly higher in the funisitis group than in the chorioamnionitis and control groups (Figure 3a,b). The mean NO concentrations in cord blood samples were higher in the funisitis group than in the chorioamnionitis group, and the mean NO concentrations were higher in both the funisitis and chorioamnionitis groups than in the control group; however, these differences only reached significance between the funisitis group and the control group (Figure 3c).

Mean BAP concentrations in cord blood samples were higher in the funisitis group than in the chorioamnionitis group. Mean BAP concentrations were higher in both the funisitis and chorioamnionitis groups than in the control group. However, these differences did not reach significance (Table 3).

#### 3.4.2. Cytokines and C5a

In the funisitis group, the concentrations of inflammatory cytokines, including IL-1b, IL-6, and IL-8, were significantly higher than in the chorioamnionitis and control groups (Figure 3d–f). However, these differences were not observed for IL-10, IFNγ, and TNFα (Table 3). Among the cytokines, only IL-6 concentrations in the chorioamnionitis group were significantly higher than in the control group (Figure 3e).

C5a was higher in the chorioamnionitis and funisitis groups than in the control group (Figure 3g).

#### 3.4.3. Correlations

In all newborns, funisitis and chorioamnionitis staging were correlated with cord blood TH (r = 0.59, *p* < 0.0001 and r = 0.64, *p* < 0.0001), BAP (r = 0.27, *p* = 0.04 and r = 0.3, *p* = 0.03), OSI (r = 0.5, *p* < 0.001 and r = 0.6, *p* < 0.001), and IL-6 (r = 0.79, *p* < 0.001 and r = 0.45, *p* < 0.001), respectively.

In cord blood, TH was correlated with BAP (r = 0.45, *p* < 0.001), and IL-6 was correlated with TH, BAP, and OSI (r = 0.69, *p* < 0.001; r = 0.39, *p* < 0.005; and r = 0.5, *p* < 0.001), respectively.

### 3.5. Comparison between Infants Admitted and Non-Admitted to the NICU

There were no significant differences in clinical characteristics between infants admitted and not admitted to the NICU in terms of their gestational age, birth body weight, gender, Apgar score at 1 min, and serum CRP. Only the Apgar score at 5 min showed a significant difference between infants admitted to the NICU and those not admitted (8.45 (8–9) vs. 8.87 (8–10), mean (range), *p* < 0.05).

Among the measured markers, serum OSI, TH, and IL-6 were higher in the babies admitted to the NICU compared with the non-admitted infants (OSI: 0.046 ± 0.005 vs. 0.026 ± 0.002, *p* < 0.001; TH: 122.6 ± 15.9 vs. 65.3 ± 5.7, *p* < 0.001; IL-6: 354.7 ± 187.5 vs. 22.35 ± 8.5, *p* < 0.05).

Multiple regression was performed to determine which of the significant markers would predict a higher risk of admission to the NICU after adjusting for the Apgar score at 5 min, being the only significant variable. Both OSI and cord blood TH were identified as significant predictors of higher risk of admission to the NICU. For OSI, *p* = 0.007, the model explained 32.5% (Nagelkerke R^2^) and correctly predicted 82.7%, and the odds of admission to the NICU were 2.3, 95% CI 1.26–4.29. For TH, *p* = 0.015, the model explained 32.8% (Nagelkerke R^2^) and correctly predicted 78.8%, and the odds of NICU admission were 1.02, 95%CI = 1.004–1.040.

## 4. Discussion

Our data showed changes in oxidative and inflammatory mediators in cord blood due to the antenatal fetal inflammatory response in full-term newborns suffering from chorioamnionitis and funisitis. Compared with newborns in the control group, newborns in the funisitis group showed significantly higher concentrations of the oxidant marker TH, but not BAP.

Higher levels of TH are reflected in higher levels of OSI, which are associated with higher levels of inflammatory cytokines, including IL-1b, IL-6, and IL-8, and more complementary activity determined by higher levels of C5a. In contrast, in the cord blood of newborns in the chorioamnionitis group, only IL-6 and C5a, which are earlier indicators of infection [13], were significantly higher than in the control group. There were no significant differences detected between the funisitis and chorioamnionitis groups in their levels of BAP and OSI, but the funisitis group had a significantly higher level of TH than in the chorioamnionitis group. These results could indicate that the fetal inflammatory response in funisitis is associated with a more severe inflammatory and oxidative reaction in the newborn than in chorioamnionitis. This was reflected in the higher incidence of admission to the NICU in the funisitis group (Table 1).

Newborns are at increased risk for oxidative stress and are more susceptible to ROS-induced oxidative tissue damage [18]. This is because newborns have weaker physiological protection against oxidation [26]. Newborns have lower circulating plasma antioxidants, such as vitamin E and beta-carotene, lower plasma levels of metal-binding proteins, such as transferrin and ceruloplasmin, and lower activity of erythrocyte superoxide dismutase compared with healthy adults [26]. Despite great research efforts, the cause(s) of the imbalance in septic and inflammatory conditions in humans remains unclear. Under normal physiological conditions, there is a homeostatic balance, which is disrupted in inflammation, between the formation and removal by elimination of endogenous antioxidant compounds [27]. Oxidative stress occurs by disrupting this balance through ROS overproduction or inadequate antioxidant scavenging defenses, and both may occur in inflammatory conditions [18,19]. The close relationship between free radicals and septic inflammatory conditions in newborns has been explained in previous studies [18,28], but the influence of ROS and antioxidants on non-septic inflammatory conditions remains unclear, particularly in newborns exposed to chorioamnionitis or funisitis. Some studies have measured oxidative parameters in patients with sepsis [27], and a few studies have examined neonates with sepsis [29], but far fewer studies have examined neonates with chorioamnionitis and funisitis.

The present work demonstrates the association of serum ROS, antioxidant potentials, and cytokines in the cord blood of newborns suffering from the fetal inflammatory response due to funisitis and chorioamnionitis. Differences in infection severity and duration were found to reflect the inflammatory state in both animal models and patients, with simultaneous concurrent changes in markers of the oxidative state [30,31]. In funisitis, inflammatory cytokines were markedly elevated, with a simultaneous increase in ROS represented by higher levels of TH and NO in cord blood.

For protection against the hazardous effects of free radicals, endogenous enzymes are provoked that specifically scavenge hydrogen peroxide, superoxide anions, and organic peroxides, followed by the repair of the damage caused in nucleic acids [15,16]. Primary antioxidants have two mechanisms of action to prevent the formation of oxygen radicals by removing their precursors or stimulating their catalysis, such as glutathione peroxidase, superoxide dismutase, and catalase. Secondary antioxidants, on the other hand, react with already formed ROS to scavenge or inhibit them, as in vitamins E and C. Endogenous antioxidants exist both intracellularly and extracellularly [27,32]. Although several reports have described antioxidant behaviors during inflammatory conditions, the exact mechanism by which antioxidants compensate for the increase in oxidant load during such conditions has yet to be evaluated widely. Circulating antioxidants include several components, such as enzymes, vitamins, beta-carotene, and proteins, and thus, cannot be evaluated as simple chemicals, and the measurement of such individual components will not provide a complete picture of the antioxidant situation in vivo. Some investigators did not report significant changes in glutathione peroxidase levels under inflammatory conditions; on the other hand, others reported a significant surge in both the activity and concentration of the enzymes catalase, superoxide dismutase, and glutathione peroxidase, and the total antioxidant capacity [19,33]. These reports suggest that elevated levels of inflammatory cytokines provoke ROS activity and production, and vice versa. Our present findings with regard to the cord blood of newborns suffering from funisitis and chorioamnionitis support this conclusion.

Some studies previously showed that ROS stimulation counteracts antioxidant production [33,34]. One pathway by which ROS oxidation provokes the activation of their counteracting antioxidants is by inducing the breakdown of the so-called Kelch-like ECH-associated protein (Keap)1 from its nuclear factor erythroid-2-related factor 2–(Nrf2)-Keap1 complex, allowing a free Nrf2 to migrate to the nucleus from the cellular cytoplasm [35,36]. Nrf2 shares in the nucleus in the activation of a series of genes coding for phase 2 enzymes by participating in their transcription factor complex, which in turn promotes antioxidant synthesis [32,34]. Our results in an in vivo study are consistent with those reports and show positive correlations between serum TH and BAP in the fetal inflammatory response, represented by their cord blood mediators.

Several reports have noted an increase in ROS in patients suffering from severe inflammatory conditions [27,31] and in animal models of septic inflammation [19,30], compared with patients with non-critical sepsis or animal controls, respectively. To our knowledge, this is the first report to show that this is also true in the fetal inflammatory response of newborns suffering from funisitis and chorioamnionitis.

FN is a more severe form of inflammation than chorioamnionitis, and the cord blood of newborns with funisitis showed higher inflammatory mediators and higher ROS, with a tendency toward higher antioxidant levels than the cord blood of newborns suffering from chorioamnionitis. This counteractive mechanism to maintain a redox state was reflected by the higher OSI in the cord blood of newborns with funisitis than in those with chorioamnionitis.

Furthermore, serum levels of OSI and TH were significant in predicting higher risk of admission to NICU when adjusted for other variables. This provides evidence that the use of serum OSI in term newborns could be a reliable indicator of the inflammatory condition, indicated by significantly higher serum IL-6 levels in admitted newborns, and could predict a higher risk of admission to the NICU.

In inflammation, the paradigm of disrupted oxidant/antioxidant homeostasis and imbalance suggests that high levels of oxidant with low levels of their counteracting antioxidant are involved in critically ill newborns [18] and patients with poor outcomes [31]. Furthermore, the oxidants became novel therapeutic targets in sepsis and inflammation [16,37]. Our results suggest that the involvement of an oxidant/antioxidant imbalance in the fetal inflammatory response to maternal chorioamnionitis and funisitis depends on the severity of the disease. This was demonstrated by the higher TH and OSI in the funisitis group and by the correlations between the funisitis and chorioamnionitis stages with their OSI and TH cord blood levels, while both stages showed a weak correlation with their BAP cord blood levels (r = 0.27 and 0.3, *p* = 0.04 and 0.03, respectively).

Evaluating the oxidative state of a critically ill newborn by measuring TH and BAP, and therefore, calculating OSI, is an easy and fast bedside technique that requires only a small amount of cord blood (60–70 µL) and is, thus, suitable for small newborns, especially premature newborns. When treating the complications of funisitis and chorioamnionitis, understanding the changes in ROS and antioxidants with the concomitant changes in inflammatory cytokines is crucial.

This is the first report to find that oxidant/antioxidant imbalances, as reflected by higher levels of oxidants, along with higher levels of inflammatory cytokines, correspond to the degree of the fetal inflammatory response to chorioamnionitis and funisitis. Furthermore, we showed that a higher level of TH in cord blood and OSI were predictive of a higher risk of admission to the NICU. Simply put, measuring cord blood TH and OSI can provide a good marker of oxidative status in critically ill newborns and predict their possible need for admission to the NICU.

## 5. Conclusions

In the cord blood of newborns with funisitis, the concentrations of TH and OSI are higher than in the cord blood of healthy newborns or newborns with chorioamnionitis. Thus, with only a small amount of the newborn’s cord blood (60–70 µL), and taking only a few minutes, measuring the level of TH and calculating OSI could be of value as an early indicator of hazardous inflammatory conditions and, hence, predict a higher risk of admittance to the NICU.

## Figures and Tables

**Table 1:** Clinical characteristics of the 52 newborns included in the study.

Item	Control *n* = 22	Chorioamnionitis *n* = 13	Funisitis *n* = 17
Female gender, *n* (%)	12 (55.5%)	7 (53.8%)	5 (29.4%)
Gestational age (weeks)	38.8 (37–41)	40 (38–42)	39.8 (37–41)
Birth (body) weight in grams, median (range)	3026 (2466–3622)	3142 (2514–3770)	3151 (2524–3682)
Mode of delivery			
Vaginal, *n* (%)	11 (50.0%)	8 (61.5%)	8 (47.1%)
Cesarean section ^a^, *n* (%)	11 (50.0%)	5 (38.5%)	9 (52.9%)
Emergency cesarean section, *n* (%)	0 (0.0%)	0 (0.0%)	4 (23.5%)
PROM ^b^, *n* (%)	2 (9.1%)	2 (15.4%)	4 (23.5%)
Maternal infection ^c^, *n* (%)	0 (0%)	1 (7.7%)	6 (35.3%) ^‡^
Admission to the NICU, *n* (%)	2 (9.1%)	3 (23.1%)	8 (47.1%) ^‡^
Apgar score (1 min), median (range)	8.3 (7–10)	8.2 (7–9)	7.5 (1–9)
Apgar score (5 min), median (range)	9.0 (8–10)	8.7 (8–10)	8.7 (8–9)
Newborn CRP ^d^ (mg/dL), mean ± SEM	0.0 ± 0.0	0.0 ± 0.0	0.56 ± 0.32 ^†^

^a^ Number of elective and emergency cesarean sections. ^b^ PROM: premature rupture of membranes. ^c^ Maternal infection was diagnosed by fever with high maternal CRP. CRP: C-reactive protein. ^d^ neonatal intensive care unit. ^‡^ Significant difference between groups, as determined by chi-square test. ^†^ Significant difference between groups, as determined by Kruskal–Wallis test.

**Table 2:** Clinical indications of cesarean section and NICU ^a^ admission for the 52 newborns included in the study.

Item	Control *n* = 22	Chorioamnionitis *n* = 13	Funisitis *n* = 17
Indications for cesarean section, *n*	11	5	9
Previous cesarean section	7	1	0
Malposition and malpresentation	3	1	2
Fetal distress or non-assuring fetal status	1	1	2
Polyhydramnios	1	1	0
Postdated pregnancy	0	1	1
Contracted pelvis and birth canal	0	2	0
Cephalopelvic disproportion	0	1	2
Maternal asthma	0	1	0
Uterine myoma	0	0	1
Pregnancy-induced hypertension	0	0	1
Intra-uterine infection	0	0	3
Indication for admission to the NICU ^a^, *n*	2	4	11
TTN ^b^	1	0	2
MAS ^c^	0	2	4
Apnea	1	0	0
Infection/sepsis	0	1	5
Hyperbilirubinemia	0	1	0

^a^ NICU: neonatal intensive care unit. ^b^ TTN: transient tachypnea of the newborn. ^c^ MAS: meconium aspiration syndrome.

**Table 3:** Concentration of interleukin (IL)-10 in cord blood, interferon γ (IFNγ), tumor necrosis factor α, and biological antioxidant potential (BAP) in the newborns studied.

Mediators	Control	Chorioamnionitis	Funisitis	*p* Value
	*n* = 22	*n* = 13	*n* = 17	
IL-10 (pg/mL)	2.49 ± 1.48	2.05 ± 1.38	3.22 ± 1.66	NS ^a^
IFN-γ (pg/mL)	0.89 ± 0.158	1.24 ± 0.28	0.765 ± 0.199	NS
TNFα (pg/mL)	0.426 ± 0.139	0.394 ± 0.087	0.410 ± 106	NS
BAP (μmol/L)	2480 ± 183	2518 ± 171	2837 ± 112	NS

Data are expressed as mean ± SEM. ^a^ NS: Non-significant.

## Data Availability

All data provided in the study are available with the corresponding author and could be provided upon request.
